# Knowledge generation towards evidence-based practice and nursing competency

**DOI:** 10.1371/journal.pone.0311285

**Published:** 2024-12-12

**Authors:** Kennedy Diema Konlan, David Adedia, Felix K. Nyande, Margaret Amenuke, Vivian Tackie, Judith A. Anaman-Torgbor

**Affiliations:** 1 Department of Public Health Nursing, School of Nursing and Midwifery, University of Health and Allied Sciences, Ho, Ghana; 2 School of Basic and Biomedical Sciences, University of Health and Allied Sciences, Ho, Ghana; 3 Department of Nursing, School of Nursing and Midwifery, University of Health and Allied Sciences, Ho, Ghana; 4 Faculty of Arts and Social Sciences, Central University, Accra, Ghana; Kwame Nkrumah University of Science and Technology, GHANA

## Abstract

**Aim:**

This study aims to describe the nursing competency, research knowledge and skills of nurses in Ghana regarding evidence-based practice.

**Methods:**

This nationwide descriptive cross-sectional survey was conducted among 480 clinical nurses and nurse educators from both private and public institutions. Nurses were included in the study if they had a full-time or part-time appointment, were in active service, and had a valid Personal Identification Number from the Nursing and Midwifery Council of Ghana. The Mann-Whitney U-test was used to compare mean rank scores between two groups, and the Kruskal Wallis H-test was used for the comparison.

**Results:**

Thirty percent of them rated their ability to formulate research questions, conduct literature search and retrieval, and critical analysis of scientific literature as fair or poor. Half of the nurses disagreed with changing to new methods, and more than half at least agreed with all the items measuring the use of evidence-based practice. One out of every 20 Nurses somewhat disagreed with formulating answerable research questions at work, and the same proportion disagreed or were neutral to integrating evidence into practice with their expertise. It was statistically significant that females (*p-value* = 0.001), married (*p-value* = 0.054), Christians (*p-value*<0.001), nurse educators (*p-value*<0.001) and part-time workers (*p-value*<0.001) were more likely to have higher mean scores on research knowledge and skills. The Kruskal Wallis test showed that research knowledge and skills (H = 97.281, *p-value*<0.001, df = 2), attitude towards evidence-based practice (H = 10.143, *p-value* = 0.006, df = 2), and nursing competencies (H = 9.041, *p-value* = 0.011, df = 2) differ for all the educational level of all nurses.

**Conclusion:**

The self-reported competencies in the various aspects of the research process and knowledge generation by both clinicians and nurse educators were good; however, the overall composite score revealed that nurses only had fair knowledge and skills in this regard. Nurses should be given the requisite training to appraise and evaluate nursing research for incorporation into nursing practice.

## Introduction

The nursing profession is distinct from other healthcare professions by its philosophy, training, approach to service delivery, and scope of practice [1]. Professional nurses’ practice in many different areas and levels; as such, they require effective clinical decision-making skills to design appropriate interventions for patient care. In the beginning, nursing practice was based on the biomedical model of care, which involved carrying out orders from physicians and focusing on treating diseases. Techniques and competencies required to practice nursing in the past were learned on the job from more experienced colleagues because very few people had any formal education [2, 3]. In Ghana, for instance, a few decades ago, only a handful of individuals who had formal education and could read and write, were taught courses like human anatomy and physiology, surgical and medical nursing, and first aid [4, 5]. Over the past few decades, the nursing profession has evolved globally, moving from total dependence on the biomedical model of care to an independent practice modality with theories, models, and distinct interventions. As the healthcare industry advances in technology and sophistication, clients’ demand for quality care is also increasing, and the nursing profession must evolve and respond to these demands.

The role of nursing education is to train nurses to develop clinical reasoning skills, which is fundamental to developing the required clinical competencies. The training curricula in most nursing training institutions in Ghana include courses such as health assessment, nursing theories, and research methods among many other courses. These courses are taught in an attempt to develop the competencies of nursing students. However, it is done largely through classroom teaching with little or no integration with clinical practice [5, 6]. This has increasingly raised concerns about nurses’ ability to integrate knowledge learned in the classroom to direct clinical/ or world perspectives [5, 7, 8]. Evidence suggests that many nurses have less confidence in their knowledge regarding EBP [7, 8], and several factors have been found to affect nurses’ knowledge and attitude toward EBP. For instance, educational level, teaching experience at different nursing levels, and additional nursing specialities have all been found to correlate positively with knowledge of EBP [1, 5, 9–11]. The level of practice is a consequence of knowledge [6]. Therefore, it is appropriate to identify the level of knowledge generation that is critical for the implementation of evidence-based practice. As a result, current research must focus on identifying nursing competencies, research knowledge and skills of nursing practice. This study therefore describes’ nursing competency, research knowledge, skills, and attitude toward EBP.

## Materials and methods

### Design

This is a nationwide descriptive cross-sectional survey and only quantitative data were collected and analyzed. The data was collected from public and private institutions, including teaching hospitals, universities, and institutions awarding diplomas in nursing.

### Study population and setting

The study was conducted among clinical nurses and nurse educators in Ghana. Clinical nurses were chosen because of the critical role they play in the health care team while the nurse educators were chosen because of their involvement in the training of the next generation of nurses. When nurse educators demonstrate good knowledge in evidence-based practice, they are likely to train students in like manner. The clinical nurses were identified from three teaching hospitals, identified based on their locations to ensure the geographical representation of nurses. Teaching hospitals are tertiary healthcare facilities that provide specialized and advanced-level care. Five teaching hospitals in Ghana provide specialized healthcare services and also serve as referral facilities for regional hospitals. The teaching hospitals in Ghana also serve as clinical attachment sites for universities to train health professionals, including doctors, nurses, and other paramedical staff. Nursing education in Ghana is done at two main levels: a diploma and a bachelor’s degree. In Ghana, universities train nurses who are awarded either a bachelor’s degree, a master’s degree, or a Ph.D. The training institutions largely train nurses awarded with a certificate (Auxiliary nurses), a diploma degree, and more recently a bachelor’s degree in some institutions. The nurse educators were identified from five universities (three public and two private) and eight nursing training colleges. Nurses were included if they had full-time or part-time appointments, were in active service, and had a valid Personal Identification Number (PIN) from the Nursing and Midwifery Council of Ghana. Auxiliary nurses, retirement/contract, were excluded.

### Sampling technique and sample size

This study used a multi-stage sampling technique. In the first stage, the teaching hospitals and the training institutions were purposely sampled based on their proportion and geographical location across the country. The entire country was zoned into three sections and nursing training institutions and teaching hospitals were selected from each section. In the second stage, professional nurses were conveniently sampled. The recruitment strategy involved the research team being present at the study sites and invited professional nurses by informing them about the study. Participant Information and Consent Forms (PICF) were provided to potential participants.

The *Raosoft* sample size calculator was used to determine the required sample size [12]. With an estimated population size of 1,500 professional nurses at the three selected teaching hospitals, with a margin of error of 5%, a confidence interval of 95%, and a default distribution of 50%, the estimated required sample size was 306 nurses. Considering non-response and withdrawal from the study, 20% (n = 61) was added to the originally estimated sample size, to get a minimum sample of 367 nurses, however, 480 nurses were recruited for this study. A population frame of all nurse educators at the training institutions was considered for this study. This is because there are not many nurse educators at the nursing training institutions in Ghana.

### Data collection instrument and data management

This study was commissioned in November 2019 and following the influence of Covid 19 and the related restrictions (social restrictions) that were imposed in Ghana, the final recruitment and complete data collection was done in July 2021. The data collection tool included closed-ended questions that were self-administered. The survey tool was adopted from previous EBP studies [13–15] with a reported reliability coefficient (Cronbach’s alpha) for each subscale exceeding 0.70 [15]. Two subscales: research knowledge, skills, and attitudes toward EBP, were adopted for this current study. There are 14 items measuring research knowledge and skills and four items measuring attitudes toward EBP. Each item was scored on a 7-point Likert scale, with one on the scale indicating poorer and 7 indicating higher knowledge and skills of EBP. The participants were asked the following: "On a scale of 1–7 where (1 = very poor; 2 = poor; 3 = fair; 4 = good; 5 = very good; 6 = excellent and 7 = exceptional), how will you rate your research skills? An additional eight items were included to assess competencies among the nurses. The nurses were asked to indicate their responses on a scale of one to seven, where one indicates "strongly disagree," and seven indicates "strongly agree."

### Statistical data analysis

The items used in measuring research knowledge, skills, and attitudes toward EBP and nursing competency in this study reported Cronbach alpha coefficients of 0.92, 0.82, and 0.87, respectively. The individual items measuring research knowledge, skills, and attitude toward EBP and nursing competencies were combined to generate a composite score for each theme by averaging. All items were positively stated except the items measuring attitude towards EBP. The data were analyzed using the SPSS software package (version 23). An initial descriptive analysis to examine participants’ responses was conducted. Basic descriptive statistics were used to summarise the data presented as mean and standard deviation for quantitative data; and median and interquartile range (IQR) for the ordinal level data. The categorical data were reported as frequency and percentages. The t-test and Mann-Whitney U-test were used to compare mean and mean rank scores between two groups, respectively, and p-values less than 5% were considered significant. Kruskal Wallis H-test was used to compare the ranked means of three groups (Nurses educated above BSc, BSc level, and below BSc) and Dunn’s test of multiple comparisons was employed.

### Data quality control

The questionnaire was pre-tested for measurement accuracy soon after the research assistants’ training. The pre-testing was done among 20 nurses at the Ho Municipal Hospital to assess the instrument’s acceptability, appropriateness of wording, and suitability. This was done to ensure that the instrument measures what it intended to measure and is suitable for its intended purposes [16, 17]. The pre-testing also gave the researchers feedback on items’ wording and responses’ clarity. The pre-testing further gave the researchers the needed experience and opportunity to administer the instruments during the main study correctly. Also, the principal researcher was directly involved in the fieldwork to ensure that the questionnaires were properly administered. In addition, the data were checked for consistency, coded appropriately, and entered into the SPSS software package for analysis. The data were explored, and general features and problem areas were displayed before data analysis commenced.

### Ethical considerations

Formal ethical approval was obtained from the University Human Research Ethics Committee (HREC) (Approval No. UHAS-REC A.1 UI 18–19). Administrative approval was obtained from all the institutions involved in this study. Potential participants were verbally invited to participate in the study, and written informed consent was obtained from the participants. The PICF explained the study’s purpose and indicated that there would be no immediate benefit for participants. It also emphasised that participation is voluntary and that deciding for or against participation or withdrawing from the study at any time will not influence any relationship with the participants.

With regards to risk, there was minimal risk associated with participation, such as loss of time. To ensure confidentiality, the participants were given unique identification numbers at the beginning of the interview. The unique codes were known to the Principal Investigator alone. All data were stored without participants. This is to protect participant identity and privacy. All comments and responses were treated confidentially. The data collected as part of this research project was stored securely as per the institutional review committee’s research data policy.

## Results

### Socio-demographic characteristics

The analysis included 480 valid responses from clinical and nurse educators and the Soci-demographic characteristics showed female (60.2%) mean age (36.62±4.66), Christian (85.20%), Diploma (39.5%), married (56.5%), and working full-time (95.0%). The detailed demographic characteristics are reported in Table 1.

**Table 1:** Sociodemographic characteristics.

Characteristics	
	Overall
	N (%)
	480 (100)
**Sex**	
Male	191 (39.8)
Female	289 (60.2)
**Marital status**	
Married	271 (56.5)
Single	209 (43.5)
**Religion**	
Christian	409 (85.2)
Moslems	70 (14.6)
Tradition	1 (0.2)
**Education**	
PhD	7 (1.4)
M.Phil.	41 (8.5)
MPH‎/MA‎/MSc	46 (9.6)
Bachelor Science	197 (41.0)
Diploma	189 (39.5)
**Work arrangement**	
Full time	456 (95.0)
Part-time	24 (5.0)
**Duration of work**	
**≤**10 years	447(93.1)
>10years	33(6.9)

Of the 480 nurses, clinical nurses had a lower mean age (30.30±4.66) than nurse educators (41.91±9.07). Most of the nurse educators (81.2%) had a postgraduate degree, whereas most of the clinicians (95.9%) had, at most, a bachelor’s degree. Clinicians were largely full-time workers (96.4%) and had at most ten years of experience.

### Research knowledge and skills

**Fig 1:**
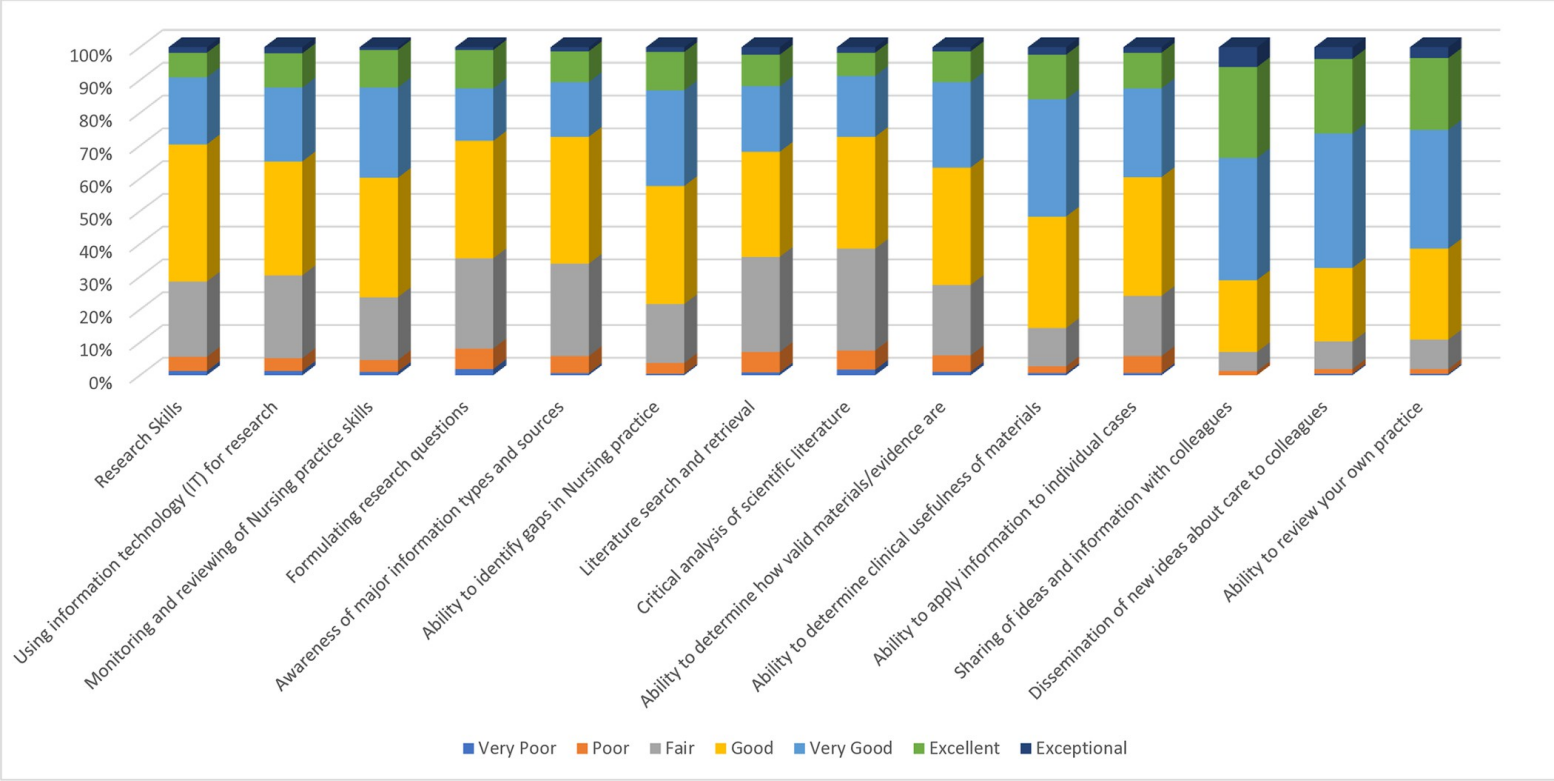
Research knowledge and skills.

A summary of the nurses’ response to the items measuring research knowledge and skill of EBP is presented in Fig 1. This result includes both clinical nurses and nurse educators.

The 14 parameters were assessed on a 7-point Likert type scale that ranged from very poor to exceptional (1 = Strongly disagree; 2 = Disagree; 3 = Somewhat disagree; 4 = Neither agree or disagree; 5 = Somewhat agree; 6 = Agree and 7 = Strongly agree). About seven out of every ten nurses indicated at least good on all the 14 items measuring research knowledge and skills. Approximately 30% rated their ability to formulate research questions, literature search and retrieval, and critical analysis of scientific literature as fair or poor. Only a few (10%) indicated an ’exceptional’ ability to share ideas and information with colleagues and disseminate new ideas about care to colleagues. The nurses’ level of research knowledge and skills for the remaining items on the scale were rated above good.

### Attitude toward evidence-based practice

**Fig 2:**
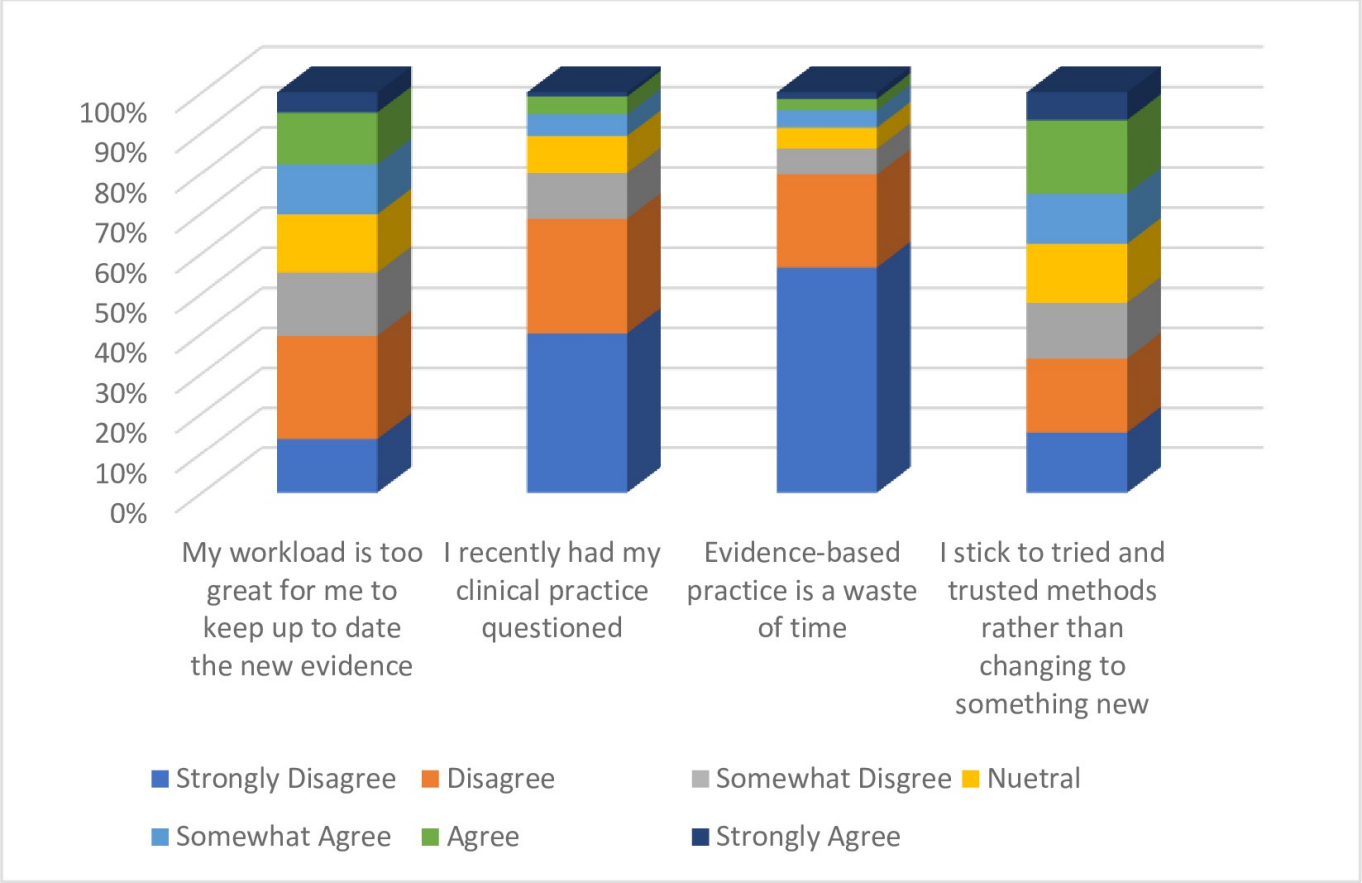
Attitude towards EBP.

Four individual statements measuring attitudes toward EBP were presented to the nurses and they were required to agree or disagree with these individual statements (Fig 2).

The attitude of nurses was measured using statements including; 1) my workload is too great for me to keep up to date with the new evidence, 2) I recently had my clinical practice question, 3) evidence-based practice is a waste of time, and 4) I stick to tried and trusted methods rather than changing to something new. These four parameters were also assessed on a seven-point type Likert scale that ranged from strongly disagree to strongly agree (1 = Strongly disagree; 2 = Disagree; 3 = Somewhat disagree; 4 = Neither agree or disagree; 5 = Somewhat agree; 6 = Agree and 7 = Strongly agree). More than half of the nurses at least disagreed with the statement: "Evidence-based practice is a waste of time"(65.0%) and "recently had my practice questioned (75%)." the nurses at least disagreed with sticking to tried and trusted methods of practice (40.0%). About five out of every ten nurses somehow disagreed that their workload was too much to keep up with new evidence.

### Predictors of research knowledge and skills

Further analysis to identify predictors of research knowledge and skills based on sociodemographic characteristics was done. The items measuring nurses’ knowledge and skills of EBP were combined to generate a composite score for the analysis (Table 2).

**Table 2:** Predictors of research knowledge and skills.

Sociodemographic Characteristics	Research Knowledge and Skill	
	Median score (IQR)	P-Value
**Sex**		
Male	4.43(3.93–5.07)	0.001
Female	4.14 (3.64–4.64)	
**Marital status**		
Married	4.29 (3.86–4.93)	0.054
Single	4.14 (3.64–4.71)	
**Religion**		
Christian	4.36(3.86–4.86)	<0.001
Islam	3.93(3.43–4.57)	
**Nurse Category**		
Clinicians	4.07(3.64–4.64)	<0.001
Educators	5.00(4.36–5.50)	
**Work-arrangement**		
Full time	4.21 (3.71–4.79)	0.003
Part time	4.68 (4.32–5.34)	
**Duration of work**		
≤10 years	4.21(3.71–4.79)	0.083
>10years	4.50(3.86–5.11)	

The median scores for research knowledge and skills for males (4.43), Christians (4.36), nurse educators (5.00), and part-time workers (4.68) were statistically significant compared to their counterparts at 0.05 significance level. The minimum level of knowledge (3.93) was recorded among the Islamic participants of the significant variables for research knowledge and skills. Overall, more than 75% of the participants had at least fair research knowledge and skills.

### Predictors of attitude towards EBP and nursing competencies

A total of four individual items were averaged to generate a composite score for the nurses’ attitudes toward EBP as well as eight items for nursing competencies. The nurses were to indicate their level of agreement with EBP statements: On a scale of 1–7 (1 = Strongly disagree; 2 = Disagree; 3 = Somewhat disagree; 4 = Neither agree or disagree; 5 = Somewhat agree; 6 = Agree and 7 = Strongly agree), indicate your level of agreement with the statement (Table 3).

**Table 3:** Attitude towards EBP and nursing competencies by sociodemographic characteristics.

Demographics Characteristics	Attitude towards EBP		Nursing competencies	
	Median (IQR)	P-Value		P-value
**Sex**				
Male	2.75(2.00–3.50)	0.371	6.00(5.50–6.44)	0.943
Female	2.75 (2.00–3.50)		6.00(5.62–6.38)	
**Marital status**				
Married	2.75 (2.00–3.50)	0.157	6.12(5.75–6.44)	<0.001
Single	2.75 (2.25–3.50)		5.88(5.50–6.25)	
**Religion**				
Christian	2.75 (2.00–3.50)	0.088	6.00(5.62–6.38)	0.411
Muslims	2.75 (2.00–3.25)		6.00(5.62–6.38)	
**Nurse Category**				
Clinicians	2.75 (2.25–3.69)	<0.001	6.00(5.50–6.38)	<0.001
Educators	2.38 (1.75–3.25)		6.26(5.97–6.53)	
**Work-arrangement**				
Full time	2.75 (2.00–3.50)	0.013	6.00(5.62–6.38)	0.893
Part time	2.25 (1.75–2.69)		6.00(5.50–6.41)	
**Duration of Work**				
≤10 years	2.75 (2.00–3.50)	0.227	6.00(5.50–6.38)	0.140
>10years	2.50 (2.00–3.25)		6.00(5.88–6.50)	

Attitude towards EBP was negatively coded; therefore, a lower score indicates a better attitude. The nurse educators (2.38) and part-time workers (2.25) have lower scores indicating a better attitude toward EBP than their counterparts. The use of EBP was significantly higher among married participants (5.50), Christians (5.33), nurse educators (5.67), and nurses who have worked for more than ten years (5.83). In addition, married participants (6.12) and educators (6.26) reported higher competencies than single (5.88) and clinicians (6.00), respectively.

### Nursing competency

**Fig 3:**
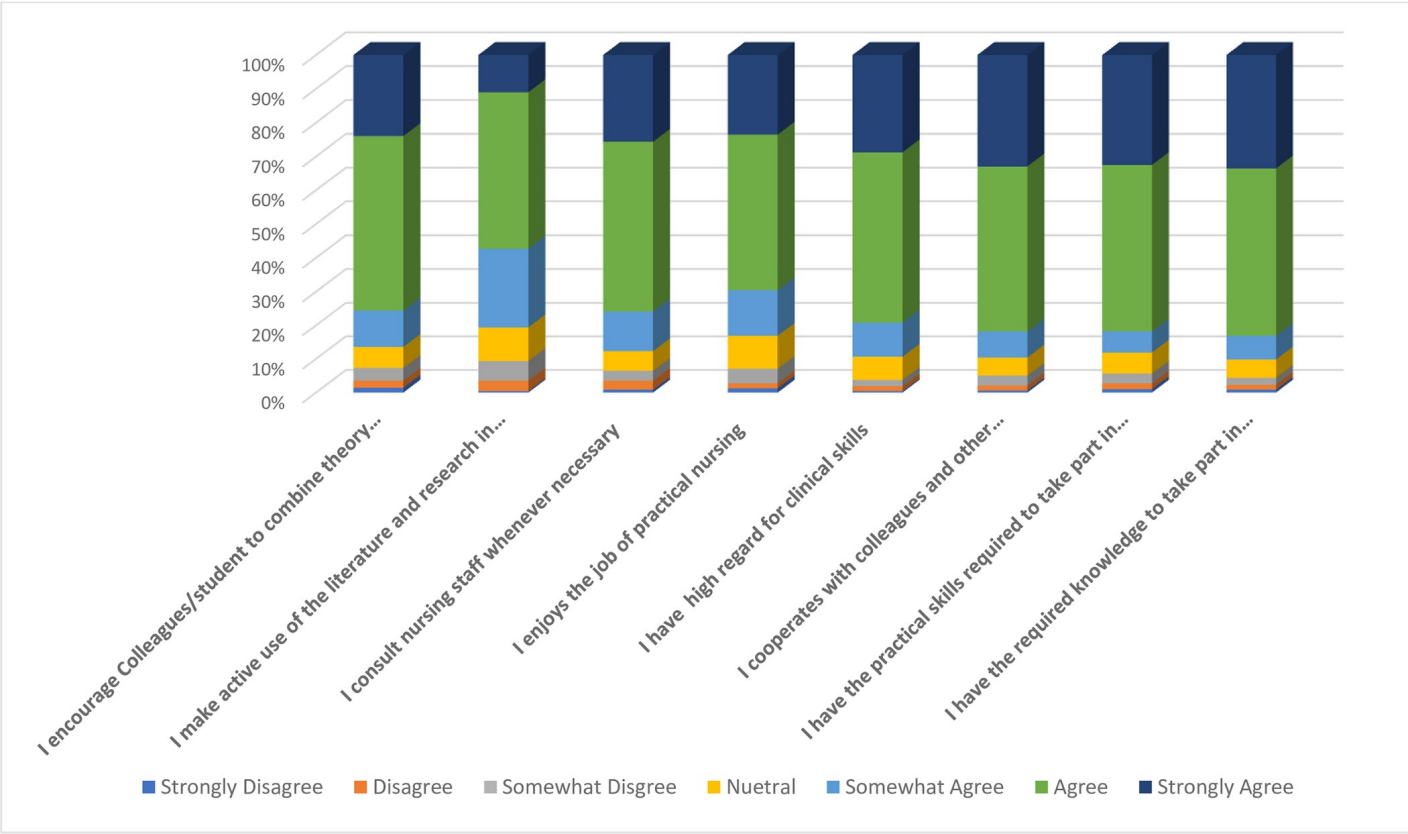
Nursing competencies.

Nursing competency was assessed among both nurse educators and clinical nurses using eight items. The nurses were asked to indicate their responses on a scale of one to seven, where one indicates "strongly disagree," and seven indicates "strongly agree." Their responses are summarised in Fig 3.

More than half of the nurses agreed with all the statements assessing their competencies, including statements such as having the knowledge and skills required to care for patients, enjoying practicing nursing, and having a high regard for clinical skills. However, 10 to 20% were neutral regarding their clinical competency, about 20% disagreed or were neutral regarding using evidence in literature in nursing practice, and also disagreed with encouraging students to combine theory and practice.

### Variable association between level of education and study variable for all nurses

The Kruskal-Wallis test (one-way ANOVA on ranks) was used to assess the association between nurses’ level of education and research knowledge, skills, and attitude towards EBP and nursing competencies. Level of education was recoded into three levels (1-above Bachelor’s Science (BSc); 2-a Bachelor’s Science and 3-below a Bachelor’s Science). The level of significance was set at *p-values*≤ 0.05 (Table 4).

**Table 4:** Association between level of education and study variable for all nurses.

	RKG/skills	Attitude to EBP	Nursing competencies
Educational level	Median (IQR)	Median (IQR)	Median (IQR)
^1^Above BSc	5.11 (4.55–5.57)	2.25 (1.50–3.00)	6.25 (6.00–6.63)
^2^BSc	4.14 (3.79–5.07)	3.25 (2.63–3.50)	5.88 (5.63–6.50)
^3^Below BSc	5.00 (4.32–5.29)	1.75 (1.63–3.00)	6.50 (5.31–6.81)
P-value*			
All	<0.001**	0.006**	0.011**
1&2	<0.001**	0.017**	0.016**
1&3	<0.001**	0.002**	0.003**
2&3	0.017**	0.305	0.552

**–Statistically Significant

RKG–ResearchKnowledge

BSc–Bachelor of Science

IQR–Inter Quartile Range

Those educated at the Bachelor of Science level have lower median scores on knowledge and skill and nursing competencies subscales. Those educated below Bachelor of Science had the lower median score on attitude to EBP, thus a better attitude towards EBP. The Kruskal Wallis test showed that Research knowledge and skills (H = 97.281, *p-value*<0.001, df = 2), attitude towards EBP (H = 10.143, *p-value* = 0.006, df = 2), and nursing competencies (H = 9.041, *p-value* = 0.011, df = 2) differ for all the educational level of all nurses. A post hoc test comparing 1) nurses educated above BSc degree and those educated at only BSc level showed statistically significant differences across all the variables, and 2) nurses educated above BSc level and below BSc level also showed statistically significant differences across all the variables. However, the comparison between nurses educated at the BSc level and those educated below the BSc level showed a statistically significant difference in only research knowledge and skills of EBP (Table 4).

## Discussions

This study described the research knowledge, skills, attitudes, and competencies of nurses and also identified the predictors of research knowledge and attitude of nurses toward using EBP. Nurses are on the frontline of service delivery in the healthcare industry more than any other health professionals, and therefore, the application of scientific knowledge in nursing service delivery is critical for practical and cost-effective ways of improving patient outcomes [3, 18]. The findings in this current study revealed a consensus among the participants about the benefits and the need to incorporate EBP into teaching and practice. However, the concepts of knowledge translation, knowledge transfer, research utilization, implementation, diffusion, and dissemination of research findings, are not well understood among the participants. These findings are consistent with previous studies [3, 19–21]. Nurses’ knowledge of EBP varies significantly across settings and categories; moreover, the level of knowledge is determined by multiple factors [5, 7, 9, 11, 21, 22]. For instance, knowledge of EBP was rated high among nurses in Oman and Kenya [23] and other health professionals in Uganda [24]. It is unclear what accounted for this contrast; however, more studies investigating nurses’ knowledge of EBP are required.

In the current study, the majority of the nurses rated their ability to formulate research questions, literature search and retrieval, and critical analysis of scientific literature as "good," while the overall composite score showed that the majority (75%) of both clinicians and nurse educators had only fair level of knowledge and skill regarding research. Self-reported ratings of knowledge and skill are higher among health professionals than objectively assessed knowledge when using the EBP scale as a discriminatory tool for the purpose of clinical certification [3, 25]. Ramírez-Vélez et al. (2015) in their study found the lack of research skills was one of the single most important barriers to EBP practice [21]. Considering that the EBP tool assessed self-reported research knowledge and skills, the tendency of the responses from the nurses in this current study to reflect a higher rating is expected; therefore, the nurses’ high rating of knowledge may not correspond with their actual clinical performance. Future studies may consider using objective forms of assessing nurses’ research knowledge and skills of EBP. Previous cross-sectional studies from Turkey and Japan reported similarly limited knowledge of EBP (e.g., clinical trial research) among nurses and varying response levels for ethical issues in research [1, 26, 27].

The disaggregated data in this current study comparing the composite score of research knowledge and skills between nurse educators and clinicians also showed that a greater proportion of the nurse educators (89.6%) have better research knowledge and skills than clinicians (58.9%). Nursing research and applying scientific knowledge are vital for clinical practice and reasoning to improve patient outcomes [6]. Research may have been on the nurse’s agenda; however, in some settings, clinical nurses were observed not to be involved in research after their formal education [22]. This calls for pragmatic training or research education for clinical nurses to be best positioned to maximise the research or scientific knowledge required for clinical reasoning. As noted by González-Torrente et al. (2012), nurses’ score on knowledge of the use of EBP was inversely related to their years of practice; that is, nurses tended to lose their EBP know-how as they stayed longer in clinical practice [28].

Overall, the nurses had a positive attitude toward EBP. Similar findings have been made by Al-Busaidi et al. (2019), who reported a high mean score for attitude towards EBP among nurses in a multi-institutional cross-sectional study in Oman [29]. Previous studies in Jordan and Norway made similar findings that nurses and nursing students valued EBP and its contribution to quality patient care; however, only a few had the self-confidence to incorporate EBP into their clinical practice [7, 23, 30]. Although the nurses are positive toward EBP, about 30% of the nurses in this current study disagreed with changing to new methods, suggesting that they are not amenable to incorporating new evidence into their practice. Also, among registered nurses in Sweden EBP capability beliefs were associated with a higher extent of EBP activities [31]. Nurses are expected to maintain professional competency, and this can be achieved by incorporating new scientific evidence into nursing practice. Therefore, there is a need for a continuous evaluation of nursing practice in Ghana. This may help in the identification of needs and the development of pragmatic interventions to advance nursing science. About 20% disagreed or were neutral regarding integrating evidence into practice with their expertise. Elsewhere, despite nurses’ positive attitude towards EBP, most nurse educators have little to average knowledge about EBP [23, 30, 32]. The management of hospitals, training institutions, and the nursing professional governing body in Ghana should consider implementing EBP training programs at workplaces and into training curricula. These training programs must target the nurse’s ability to formulate the research question, literature search, retrieval, and critical analysis of scientific literature.

The study identified the category of a nurse (nurse educator or clinical nurse), nature of employment (full-time versus part-time), marital status, religion, and longer duration of work to be the determinants of attitude towards and use of EBP. Nurse educators and part-time nurses were also found to have a more positive attitude toward EBP than clinicians and full-time nurses. This could be because nurse educators were likely to have higher academic qualifications and be exposed more to research and literature than their clinician counterparts. These findings support a study by González-Torrente et al. (2012), also in their study among Spanish nurses, found the duration of practice to be associated with knowledge of EBP [28]. However, the duration of the study was inversely related to the knowledge of EBP. Previous studies showed that certain categories of nurses (gerontological nurses) were more likely to practice EBP than others [31].

### Implication for nursing practice

Nurses require clinical reasoning; a cognitive process of applying knowledge and experience to the clinical decision-making process in the 21st century to deliver specialised nursing services with a high degree of professionalism and optimum patient outcomes. The current findings have both practical and theoretical implications. Practically, the use of EBP in the care of patients is likely to be affected since the core concepts of knowledge translation, knowledge transfer, research utilization, implementation, and diffusion, as well as the dissemination of research findings, are not well understood among nurses. These nurses are not likely to incorporate EBP into their practice. Similarly, the lack of appreciation of these core concepts of EBP could affect the teaching of EBP to students as well as the use of EBP to build a knowledge base to support the science of nursing.

### Strength and limitations

One important strength of the current study is the use of large sample size (480) of nurses. This was because research assistants actively stayed within healthcare institutions and related facilities to encourage participants (through persistent follow-up) to respond to the survey. This study was limited to only 3 teaching hospitals, 5 universities, and 8 nurses and midwifery training institutions hence, generalization of the study findings should be done with caution as it may not be a general representation of the Ghanaian situation. However, the findings provided a cursory picture of the situation of evidence-based nursing in the Ghanaian context. The limitations of the study are that clinical nurses included in the study were recruited from teaching hospitals and did not include nurses from lower-level and private hospitals. These categories of nurses could have added another perspective to the study. However, it is noteworthy that teaching hospitals have the country’s largest and most diverse categories of clinical nurses. Again, the sampling of nurse educators from nursing training colleges is limited to a few institutions. Another critical limitation of this study was the lack of use of objectively verified tools to assess evidence-based practice of nurses. We think that future research should incorporate the use of objective research tools to evaluate evidence-based practice. However, this study highlights the situation and the attributing factors influencing the generation of knowledge for evidence-based practice in Ghana.

## Conclusion

Adequate knowledge, skills, and the right attitude toward research are essential in the uptake and incorporation of research findings in nursing practice and nursing education. The study’s findings revealed a disparity between self-reported research competence and the overall composite score regarding research knowledge and skills among nurses. The self-reported competencies in the various aspects of the research process and knowledge generation by both clinicians and nurse educators were found to be good; however, the overall composite score revealed that nurses only had fair knowledge and skills in this regard. Thus, the composite score did not corroborate the self-belief of the nurses regarding their research competencies. Comparatively, nurse educators were found to have higher research knowledge and skills of EBP relative to clinical nurses. The knowledge gap between nursing education and clinical nurses must be bridged to facilitate EBP and advance nursing science in Ghana. Further research is required to investigate the barriers to EBP among nurses.
